# COVID-19 non-vaccination among older adults in China: a nationwide survey based on the China Health and Retirement Longitudinal Study (CHARLS)

**DOI:** 10.21203/rs.3.rs-2380496/v1

**Published:** 2022-12-19

**Authors:** Gewei Wang, Yao Yao, Yafeng Wang, Jinquan Gong, Qinqin Meng, Hui Wang, Wenjin Wang, Xinxin Chen, Yaohui Zhao

**Affiliations:** 1. Institute of Social Science Survey, Peking University, Beijing, China.; 2. China Center for Health Development Studies, Peking University, Beijing, China.; 3. China Center for Economic Research, National School of Development, Peking University Beijing, China.

## Abstract

China has a lower rate of vaccination among older adults and those who have chronic conditions and functional disabilities. As China has recently ended the zero-COVID policy, understanding the factors behind low vaccination rates among these vulnerable populations can inform immediate policy priorities to save lives for China and offer lessons for the world at large. We used the fifth wave (2021–22) of the China Health and Retirement Longitudinal Study (CHARLS), which represented mainland Chinese 45 and older. Vaccination status was updated in the summer of 2022, reflecting the current situation because very few additional vaccinations were administered afterward. For those who were unvaccinated, self-reported reasons were recorded. Using regression analysis, we investigated the determinants of non-vaccination, including demographics, functional status, and chronic conditions. In addition, two-thirds of the respondents had their vaccination status recorded twice in 2021 and 2022, allowing us to examine changes in vaccination rates in the recent year, zeroing in on the effects of the government’s most recent vaccination campaign. Finally, we corroborated the regression results using self-reported reasons for non-vaccination in both years. A total of 12900 participants were included in the analysis. By the summer of 2022, the weighted COVID-19 vaccination rate among older Chinese people (≥60 years old) was 92.3%, with 88.8% having completed the primary series and 72.7% having received boosters. Only 72.0% of the oldest-old (≥80 years old) had completed the primary series, and 47.1% had had boosters. Regression analysis showed that participants who were older, female, unmarried, registered with urban Hukou residence, functionally dependent, and comorbid with chronic conditions were less likely to receive COVID-19 vaccines. A significant increase in vaccination rates among ethnic minorities, older adults, rural residents, and those with chronic conditions and functional dependency was observed in the year after the winter of 2021 when the government started to push for universal vaccination. The self-reported reasons for non-vaccination in 2022 were contraindications (48%), advanced ages/frailty/health conditions (21%), problems in accessing vaccines (18%), concerns about side effects or efficacy (9%), and having never heard of COVID-19 vaccine (6%). Nevertheless, as China has ended the zero-COVID policy, many older people, especially the oldest and those with chronic conditions and disabilities, have not yet been fully vaccinated with the primary series or booster doses, exposing them to the danger of infection. Therefore, health authorities should immediately abandon the previous practice of refusing to vaccinate those with chronic conditions, change people’s mistaken perceptions of contraindications and side effects, and improve access to vaccines. Most importantly, China should strengthen public trust in vaccines by making information transparent regarding the vaccine’s protection rates and side effects.

Vaccination is proven to be the primary defense against COVID-19, which has killed over 6.6 million people worldwide to date, mostly older adults.^1,2^ Much of the deaths occurred in the initial phase of the pandemic when vaccines were not available. However, even after vaccines became freely available, a substantial proportion of older people from various countries were reluctant to take them.^3^ Studies have demonstrated that vaccines effectively reduce critical conditions and fatalities.^3^ Therefore, vaccinating older populations is of critical importance.^4,5^

China has the largest number of older individuals globally.^6^ Fearing the catastrophe for the older population under a less developed medical infrastructure, China had adhered to a zero-COVID strategy in the past three years, effectively shielding the population against COVID-19. However, the highly infectious Omicron variant severely challenged this strategy by raising the economic and social costs of containment. As infections and quarantines skyrocketed in November, squeezing people and the government to the breaking point, the Chinese government finally ended the zero-COVID policy. As of December 11, 2022, most cities have stopped requiring COVID tests in public places, and those infected are no longer subject to mandatory quarantines, practices that China had adhered to for nearly three years.

What does the about-face imply for vulnerable older people? Official statistics have consistently shown lower vaccination rates among older Chinese than their younger counterparts, contrary to the pattern in the United States and other neighbouring countries, and this had been the stated reason for adhering to the zero-COVID policy despite being the last major country to relax restrictions.^6,7^ It is thus intriguing why China lagged in vaccinating its older population. Answers to the question can also inform what China can and should do to reverse the situation.

Before delving into data analysis, we first gain insight by reviewing China’s vaccination policies. When the China-made vaccines first became available in the spring of 2021, China excluded older populations (60+) from vaccination, citing fears of potential side effects. Older people were permitted vaccination a few months later. Even then, for a long time, vaccinators routinely turned away people they suspected might be in danger of side effects. In particular, anyone older than 60 would first receive a blood pressure test and be disqualified if the diastolic pressure went above 160. The vaccinators would also ask about diabetes, heart diseases, strokes, kidney conditions, cancer, etc., and refuse those at risk.^8^ The definition of contraindication was broad and ambiguous, which also caused public distrust of the vaccines. In the third quarter of 2021, as the Delta variants rampaged through India, China introduced booster shots for younger adults and approved and promoted vaccination among children. As Omicron became the dominant variant in late 2021, efforts were made to vaccinate as many people as possible. The measures included financial incentives, material gifts, coercion in some places, and vaccination vans driven directly into communities to provide residents convenience. Free insurance against adverse reactions was also provided. However, vaccinators continued to exclude people they suspected of having contraindications. Finally, on November 29, 2022, following the abrupt COVID policy change, a new guideline was released, emphasizing the importance of completing primary series and boosters in older populations.^9^ The various stages of vaccination and vaccination rates among older people in China are displayed in Panel 1.

Developing immediate and efficient vaccination programs requires understanding the determinants, such as the concerns and motivations influencing the decision-making for vaccination among older people. The above policy review suggests that China’s delay in vaccinating older people and those with chronic illnesses was possibly not due to age discrimination but ill-placed paternalism centred around the fear of side effects.

Vaccination resistance and hesitation are common among older people in many countries.^10–13^ A study of older Europeans found age and chronic conditions positively associated with vaccination.^13^ The difference between China and Western countries suggests the role of health authorities deserves special attention, especially in how they approach vaccine side effects. Research is lacking in this area. A few papers have studied vaccine uptake in Hong Kong or parts of China, but none has examined the role of health issues, and none was based on a nationwide community-dwelling older population in China.^14–16^

To address the aforementioned gaps, we included vaccine questions in the fifth wave of the China Health and Retirement Longitudinal Study (CHARLS) in the summers of 2021 and 2022, with vaccine status updated for all respondents in the summer of 2022. Since the vaccination rate did not increase between the summer and November 2022 (shown in Panel 1), our data reflect the current vaccination status among older adults in China. We investigated the determinants of not vaccinating by regression and textual analysis of narrative reasons. Our recommendations for the vaccination program were based on evidence from the above qualitative and quantitative analysis.

## Results

A total of 12900 participants were included in the analysis of vaccine status as of the summer of 2022. The mean age of the participants was 65.2±9.5 years old [range from 52 to 101], with 53% being female. The majority of the participants were married (82.6%), Han (93.0%), and rural (81.4%). 13.2% of the total had functional dependency (13.2%), 44.9% had doctor-diagnosed hypertension, and 21.9% had doctor-diagnosed diabetes. The unweighted non-vaccination rate of the participants was 6.1%, varied from 3.7% in the 52–59 age group to 17.9% in the oldest-old group aged 80 and over. A detailed description of the individual characteristics of study participants is presented in Table 1.

The weighted vaccination rate (having received at least one dose) for older people (aged 60+) was 92.3%; among the vaxers, the completion rates of primary series and booster doses were 88.8% and 72.7%, respectively (Figure 2). Vaccination rates declined with age. Among the oldest-old people (aged 80+), the weighted rates for first-dose, primary-series, and booster vaccinations were 80.6%, 72.0%, and 47.1%, respectively.

The factor associations with vaccination status in logistic regressions are shown in Table 2. We focused our attention on receiving at least one dose because they were likely to have overcome hesitation and were given the green light by the doctor and, given time, were likely to go on for the second and boosters. Female, unmarried, and older adults registered with urban Hukou were associated with a lower chance of receiving at least one dose (*P*s<0.05). Among the groups by age, controlling for other factors, the oldest-old group (80+) was the least likely to receive vaccination than those aged 52 to 59 years (OR=0.37 [0.28–0.49], *P*<0.001). Those who were functionally dependent were less likely to receive vaccines than those who were independent (0.29 [0.24–0.35], *P*<0.001). A similar pattern was observed in patients with certain chronic conditions that are often mistakenly regarded as contraindications, including hypertension (0.84 [0.72–0.99], *P*=0.037), heart disease (0.61 [0.51–0.74], *P*<0.001), stroke (0.75 [0.58–0.96], *P*=0.023), diabetes (0.79 [0.65–0.98], *P*=0.029), lung disease (0.71 [0.57–0.88], *P*=0.002), and cancer (0.25 [0.18–0.34], *P*<0.001). However, kidney and liver problems (cancer excluded) or dyslipidemia did not have statistically significant effects on vaccination, presumably because they were not considered contraindications. Interestingly, those with arthritis were more likely to be vaccinated, suggesting that people with mild chronic diseases with no chance of side effects wanted protection. Digestive diseases were another such example: they were positively associated with vaccination, and the statistical significance was marginal in the single dose equation but strongly significant in complete vaccination and boosters.

When primary series and booster vaccinations were considered, the directions of the associations, particularly with age, functional dependency, and chronic diseases, remained the same. Because of delays in vaccinating older individuals, their odds of having complete and booster shots were significantly lower than the first dose. The same went for non-Han ethnicity.

There were no significant differences in any vaccination status by educational level.

In the multinomial logit regression with three vaccination outcomes (never vaccinated, vaccinated before the summer of 2021 - Phase 1, and afterward – Phase 2), we examined the vaccination timing (Table 3). Non-vaccination was used as the base outcome in Columns 1 and 2. During Phase 1, when COVID-19 vaccines had only been available for six months, female, unmarried, widowed, non-Han, urban, older, functionally dependent, and chronically ill adults had lower odds of receiving vaccinations (Ps for RRR < 0.01). Phase 2 showed significant improvements, especially for older groups, those with functional dependency, and those with major chronic diseases that were possibly considered contraindications, reflecting the government’s efforts to narrow the deficits via mobile vaccination vans and providing financial incentives for vaccinations.

The changes in priorities are more clearly shown in Column 3, where we presented the relative odds ratio between late and early vaccinations. Those who were initially neglected but caught up in a year were ethnic minorities (RRR=2.39 [1.92–2.97], P<0.001), ages 60–69 (2.20 [1.85–2.61], P<0.001), 70–79 (3.35 [2.78–4.03], P<0.001), and particularly aged 80 and older (8.44 [6.65–10.72], P<0.001). Similarly, those with functional dependency (2.41 [2.08–2.79], P<0.001) and major chronic conditions considered to be contraindications experienced large and significant increases: hypertension (1.30 [1.16–1.45], P<0.001), stroke (1.53 [1.25–1.87], P<0.001), diabetes (1.21 [1.04–1.42], P<0.001), and cancer (2.20 [1.60–3.03], P<0.001).

Sex differences did not change between the two phases (1.03 [0.93–1.13], *P*=0.605), suggesting the persistence of women lagging behind. The vaccination rates of married older adults were drawn closer to their counterparts in Phase 2 (0.84 [0.72–0.97], *P*=0.02), but this catching-up effect was small. Urban older adults fell behind even further during Phase 2 (0.70 [0.59–0.84], P<0.001). The urban-rural difference probably reflected much higher employment rates among urban residents, which subjected them to mandatory vaccine requirements in workplaces.

Because the oldest-old were almost neglected in phase 1 (0.19 [0.14–0.26], P<0.001), such a large ramping-up hadn’t achieved the same coverage for the oldest-old as for other age groups at the end of phase 2. but at the end of phase 2, they were still under-vaccinated.

It is worth noting that despite the latest vaccine campaign targeting older individuals, which narrowed the vaccine deficits among vulnerable groups, older people with functional dependency and major chronic conditions remained less vaccinated, as we demonstrated earlier in Table 2.

Table 4 showed the categorised reasons for non-vaccination by the end of each of the two phases. By the summer of 2022, the top five reasons were having contraindications (48%), being old/ frail/with health conditions (21%), accessibility to vaccination due to problems like travel, mobility, and short supply (18%), concerns about the side effect or efficacy (9%), and having never heard of COVID-19 vaccine before (6%). Among the oldest-old, lack of knowledge about vaccines was an important issue. In contrast, concerns regarding contraindications were more pronounced among younger-old participants (Appendix Table A4, pp5). More concerns were voiced in phase 2 on health issues. We observed a substantial increase in reasons attributed to contraindications, from 30% by the summer of 2021 to 48% in the next year. Reductions are observed for reasons citing having access problems (from 22% in 2021 to 18% in 2022) and being unaware of the vaccine (from 8% in 2021 to 6% in 2022) among the entire older population.

## Discussion

Despite the Chinese government’s successful campaign that increased vaccination rates among older people, the effort is insufficient. It is still the case that vaccination rates among older people remain lower than those among their younger counterparts and lower than those in many neighbouring countries. Only 72.0% of the oldest-old (80+) have completed their primary series, and 47.1% received booster doses as China abandons the zero-COVID policy.

COVID-vaccination resistance and hesitation are common among older people in many countries.^10–13^ Using both quantitative and qualitative analyses, we found that women, older adults, unmarried or widowed, urban, functionally dependent individuals, and those with chronic diseases were less likely to receive COVID-19 vaccinations. Many of the above results conformed to the existing international literature on vaccine uptake. For example, other studies from China and France also observed a higher vaccination resistance among women, who tend to have greater fears of vaccines.^17,18,19^ The urban-rural difference probably reflected much higher employment rates among urban residents, which subjected them to mandatory vaccine requirements in workplaces.^20^ Unmarried or widowed older individuals, often called ‘the hidden elderly,’ had lower vaccination rates, possibly because of information deficits or lacking social support. ^21,22,23^ In many other countries, people with functional disabilities are shown to be less likely to receive vaccinations; therefore, policies often target this group by providing in-home vaccinations.^24^ A study from Shanghai, China found that people older than 60 years of age who were comorbid with diabetes had lower booster vaccination rates than those younger than 60 years of age.^7^ We are the first to show that urban residents have lower vaccination rates than their rural counterparts, probably because of urban residents’ lower employment rates or their lower response to financial incentives or compliance with coercive measures.

However, China stands out for the negative effects of advanced ages and chronic illnesses on vaccination. These are particularly dangerous. Given the paternalistic tradition in China, the way the health authorities handled information about vaccine side effects seemed to have played a role. In particular, vaccine clinics have refused to vaccinate older people with potentially life-threatening conditions. The practice has continued albeit lessened since the vaccine push started to target older populations with chronic illnesses in late 2021. This improper behaviour undermined people’s trust in vaccines.

A major strength of this study is the relatively precise estimation of up-to-date vaccination rates among older people by taking advantage of the nationally representative cohort of older adults in China. We reported the uptake rates for receiving at least one dose, primary series vaccination and having received booster doses in the summer of 2022, after which the vaccination rate in China remained stagnant. A second strength of the study is the successive surveys of the study sample in the summers of 2021 and 2022, which enabled us to track the dynamics in vaccination among diverse subpopulations and to identify the role played by health authorities in this process. Furthermore, by combining the qualitative and quantitative analysis, we examined the determinants of non-vaccination among older adults, allowing us to formulate timely and pragmatic policy recommendations for the next vaccination campaign.

This study has some limitations. In particular, for respondents who completed interviews in 2021, although their vaccination status was updated to 2022, the rest of the questionnaire was not. Therefore, a timing mismatch by one year exists for a subset of respondents. We think this problem is not serious because chronic conditions rarely change within one year. Nevertheless, we conducted a robustness analysis using chronic conditions derived from the 2018 wave for all respondents, and the results remained unchanged (Appendix Table A2 and A3, pp 4–5).

Our study supports the need for an immediate vaccination and information campaign tailored to older people, particularly those over 80 and comorbid with multiple chronic conditions. China does not have time to lose. Major cities have already shown high infection levels; the outbreak will soon spread to smaller cities and vast rural areas. The National Health Committee has outlined specific strategies to improve vaccination rates among older people.^9^ In December 14, the health authority initiated the second booster shot for populations with high transmission risk, aged 60 years and older, comorbid with severe chronic conditions, and with compromised immune systems.^25^ To effectively implement these strategies, China should quickly restore people’s trust in COVID-19 vaccines and fight misinformation by providing transparency. In particular, statistics on adverse vaccine responses should be revealed. Meanwhile, the government should immediately start another vaccination campaign and use the measures that were previously proven effective, especially running vaccine vans for communities and in-home vaccinations for the disabled.

Dispelling the misinformation also requires concerted efforts by physicians, healthcare providers, family members, and others who are trusted by older people. The necessity of vaccination, the side effects, and the list of contraindications may be advertised by influential physicians and celebrities. Their advocacy will enable older populations who lack health literacy and are digitally excluded to weigh the pros and cons of vaccination to make an informed decision.

## Methods

### Study design and participants

CHARLS is a longitudinal study that surveys a nationally representative sample of middle-aged and older Chinese. The baseline survey in 2011 covered 150 counties or districts and 450 villages or communities by multistage stratified random sampling and interviewed a total of 17708 individuals from 10257 households, all face-to-face in their places of residence with computer-assisted personal interviews (CAPI). Details of sampling strategy are reported in Supplementary Methods (pp.2). CHARLS respondents have since been reinterviewed as part of the regular waves in 2013, 2015, 2018, and 2021/22.^26^ By 2021/22, CHARLS remained representative of Chinese 52 years and older.

The fifth wave of CHARLS, including a module on COVID-19, was originally planned for the summer of 2021, but widespread restrictions disrupted fieldwork due to COVID outbreaks. 11 647 individuals were eventually interviewed in this phase. In phase 2, the summer of 2022, as the survey resumed with visits of 3 646 individuals not interviewed in phase 1, we attempted to revisit all sample individuals to update their vaccination status. The number of completed sample respondents was 15 293.

To adjust for sample attrition, we constructed a Probit model to account for attrition and multiplied the inverse of the predicted response probability with original sampling weights inherited from the previous wave to derive the updated sampling weight. We further aligned the sample with the age-sex composition in national population statistics and derived post-stratification weights for sample respondents aged 52 and over (n=14 570), among which 1 670 individuals did not respond to the vaccine questions in 2022, leaving the sample size for our analysis to 12 900.

**Figure 1.**
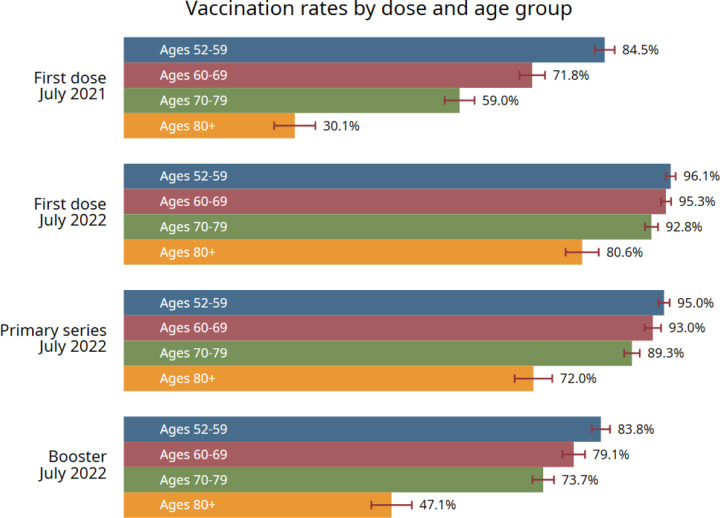
Vaccination rates among older Chinese Note: 95% confidence intervals are shown as capped, horizontal spikes.

**Figure 2.**
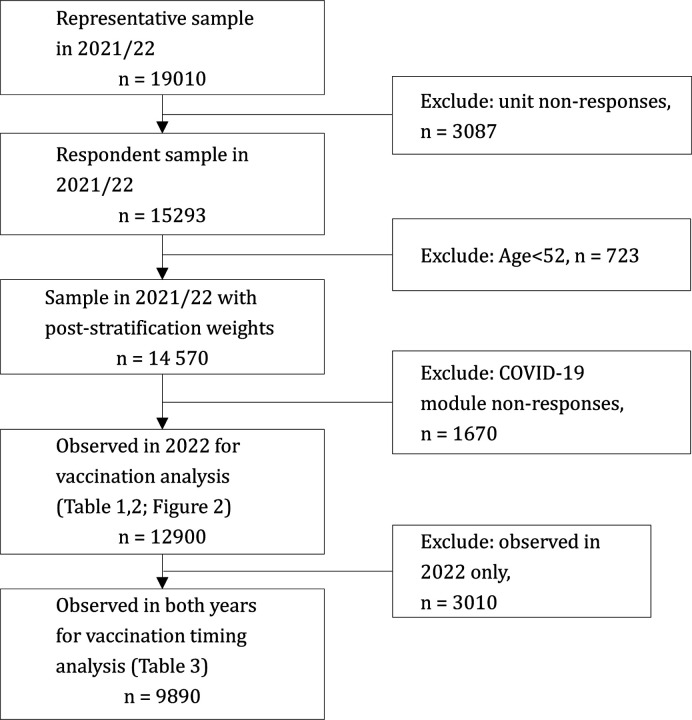
Flow diagram of this study

Vaccination-related questions asked about the number of doses they had received, the number of required doses for completing primary series vaccination, and reasons if still being unvaccinated. A subset of 9 890 individuals answered the COVID-19 module in both phases, which allowed us to use this sample to study vaccination timing for the first dose. Sample inclusion is described in the diagram of Figure 1.

All participants or their legal representatives signed written informed consent forms to participate in the baseline and follow-up surveys. Informed consent was obtained from study participants before completing the study questionnaire. The study was approved by the Biomedical Ethics Committee of Peking University, Beijing, China (IRB00001052–11015).

### Procedures

The COVID-19 vaccination rate is defined as the share of those having received at least one dose of COVID-19 vaccines by the time of interview in the summer of 2022. Depending on whether the number of doses received is less than, equal to, or more than the dose requirement of primary series vaccination, we further divided the vaccinated into three groups: incomplete vaxers, primary series vaxers, and booster vaxers.

CHARLS data included basic socio-demographics, including age, sex, marital status, ethnic origins, education, Hukou residence of rural or urban household registry, and regional residence. We divided the sample into four age groups, aged 52–59, 60–69, 70–79, and above 80. Marital status was classified as married and unmarried (single or widowed). Ethnicity contained Han and other minorities referred to non-Han. Education attainment was divided into three groups: illiterate, literate who had not finished primary school or completed primary school, and those who had completed middle school or more. The regional residence follows the official definition of four regions: northeast, east, middle, and west.

Health measures included 12 diagnosed chronic conditions reported by the interviewees. We used self-reported conditions because the vaccination decision would be based on the information known to the respondent. We use the ability to perform six basic activities of daily living (ADLs) and five instrumental activities of daily living (IADLs) to indicate the travel accessibility to vaccine clinics. The variable “ADL/IADL dependency” is defined as needing help performing any activities.^26^ For each of these conditions, we constructed a binary indicator, 1 for having the condition, 0 for not having the condition.

The unvaccinated interviewees also offered reasons in great detail. We conducted a textual analysis by searching for keywords, defined 20 reasons for being unvaccinated, and further classified into nine categories.

### Statistical analysis

We calculated vaccination rates at the national level and among demographic groups, and also provided statistics for primary series vaxers and booster vaxers. We calculated the share of each category of self-reported reasons among those unvaccinated.

To examine vaccination factors, we fitted a logistic regression to model the vaccination decision:

(1) $$\mathrm{Pr}\left(y_{i}=1|x_{ij},j=1\ldots k\right)=\frac{\exp\left(\beta_{0}+\sum_{j=1}^{k}\beta_{j}x_{ij}\right)}{1+\exp\left(\beta_{0}+\sum_{j=1}^{k}\beta_{j}x_{ij}\right)}$$

in which *y*_*i*_ is a binary indicator of whether a person *i* has received at least one dose as of the summer of 2022, *x*_*ij*_’s are the *j* explanatory variables in regression as the sociodemographic factors and health measures. We estimated standard errors by clustering the sample at the household level to correct for within-household correlations. The same model was applied in studying the decision to complete primary series vaccination and booster shots.

To maintain the same sample between the descriptive and regression analysis, we imputed a few tens of missing sociodemographic factors, chronic disease incidence, and ADL dependencies (Appendix Table A1, pp 3).

We examined the dynamics of vaccinations by analysing the twice-interviewed personal vaccination status collected in the summers of 2021 and 2022. As Panel 1 shows, there were two waves of vaccine campaigns. The first was in the middle of 2021, aiming at the entire adult population. The second wave started in October 2021 and lasted for another half a year, focusing more on older adults. These two waves coincided with the two phases of the CHARLS 5^th^ (2021/22) wave, so we constructed a multinomial logit regression model to study whether a person’s first-dose vaccination occurred in phase 1, phase 2, or neither. The comparison of vaccination outcomes between the two phases can shed light on the priorities of vaccination policy.

The regression framework of multinomial logit is specified as follows:

(2) $$\mathrm{Pr}\left(y_{i}=n|x_{ij},j=1\ldots k\right)=\frac{\exp\left(\beta_{0}^{\left(n\right)}+\sum_{j=1}^{k}\beta_{j}^{\left(n\right)}x_{ij}\right)}{1+\exp\left(\beta_{0}^{\left(2\right)}+\sum_{j=1}^{k}\beta_{j}^{\left(2\right)}x_{ij}\right)+\exp\left(\beta_{0}^{\left(3\right)}+\sum_{j=1}^{k}\beta_{j}^{\left(3\right)}x_{ij}\right)}$$

where *β*^(1)^ = 0. We specify three outcomes: unvaccinated (*y*_*i*_ = 1), vaccinated at phase 2 (*y*_*i*_ = 2), or vaccinated at phase 1 (*y*_*i*_ = 3). Explanatory variables are the same as in the logistic model for the binary choice. The value of this multinomial model lies in the comparison of factor associations with late vaccination relative to early vaccination.

A robustness check was conducted to replace the health variables with measures in wave 2018, which could represent the beginning-of-period status for regression (Appendix Table A2, pp 4). Our results remained the same.

All statistical analyses were performed with Stata version 17.0.

## Supplementary Material

Supplement 1

## Figures and Tables

**Panel 1.**
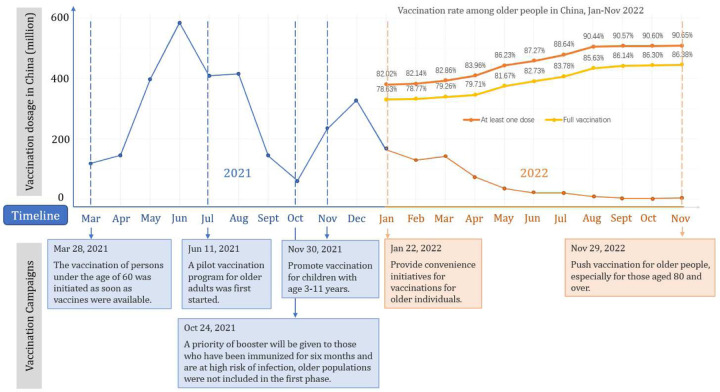
Vaccination campaign and the vaccination rate among older adults in China

**Table 1.** Characteristics of study participants of CHARLS, by age groups

	Total sample n=12900	52–59 n=3536	60–69 n=4587	70–79 n=3578	80 and + n=1199
**Sex**					
Female	6858 (53.2%)	1919 (54.3%)	2429 (53.0%)	1858 (51.9%)	652 (54.4%)
Male	6042 (46.8%)	1617 (45.7%)	2158 (47.0%)	1720 (48.1%)	547 (45.6%)
**Marital status**					
Married	10635 (82.6%)	3290 (93.2%)	4047 (88.4%)	2701 (75.5%)	597 (49.9%)
Unmarried or widowed	2245 (17.4%)	241 (6.8%)	529 (11.6%)	875 (24.5%)	600 (50.1%)
**Vaccination status**					
Unvaccinated	791 (6.1%)	130 (3.7%)	199 (4.3%)	247 (6.9%)	215 (17.9%)
Only one doze	337 (2.6%)	43 (1.2%)	85 (1.9%)	115 (3.2%)	94 (7.8%)
Only two doses	1805 (14.0%)	379 (10.7%)	587 (12.8%)	560 (15.7%)	279 (23.3%)
Booster	9967 (77.3%)	2984 (84.4%)	3716 (81.0%)	2656 (74.2%)	611 (51.0%)
**Ethnicity**					
Han	11929 (93.0%)	3231 (92.1%)	4230 (93.0%)	3342 (93.7%)	1126 (94.1%)
Non-Han	892 (7.0%)	276 (7.9%)	320 (7.0%)	225 (6.3%)	71 (5.9%)
**Education**					
Illiterate	3716 (28.8%)	539 (15.2%)	1310 (28.6%)	1291 (36.1%)	576 (48.0%)
Primary (include literate)	4899 (38.0%)	1392 (39.4%)	1456 (31.7%)	1621 (45.3%)	430 (35.9%)
Middle and high school +	4281 (33.2%)	1604 (45.4%)	1820 (39.7%)	664 (18.6%)	193 (16.1%)
**Residence (Hukou)**					
Rural residence	10500 (81.4%)	2963 (83.8%)	3773 (82.3%)	2841 (79.4%)	923 (77.0%)
Urban residence	2398 (18.6%)	572 (16.2%)	814 (17.7%)	736 (20.6%)	276 (23.0%)
**Region**					
East	4448 (34.5%)	1215 (34.4%)	1705 (37.2%)	1116 (31.2%)	412 (34.4%)
Central	3836 (29.7%)	1034 (29.2%)	1301 (28.4%)	1134 (31.7%)	367 (30.6%)
West	3719 (28.8%)	1050 (29.7%)	1218 (26.6%)	1096 (30.6%)	355 (29.6%)
Northeast	897 (7.0%)	237 (6.7%)	363 (7.9%)	232 (6.5%)	65 (5.4%)
**Functional dependency**	1699 (13.2%)	226 (6.4%)	464 (10.2%)	601 (16.8%)	408 (34.2%)
**Chronic conditions**					
Hypertension	5773 (44.9%)	1198 (34.0%)	2013 (44.0%)	1937 (54.2%)	625 (52.4%)
Heart diseases	2814 (21.9%)	522 (14.8%)	979 (21.4%)	999 (28.0%)	314 (26.3%)
Stroke	809 (6.3%)	122 (3.5%)	248 (5.4%)	329 (9.2%)	110 (9.2%)
Diabetes	1980 (15.4%)	442 (12.5%)	757 (16.6%)	629 (17.6%)	152 (12.8%)
Lung diseases	1975 (15.4%)	381 (10.8%)	684 (15.0%)	671 (18.8%)	239 (20.1%)
Cancer	331 (2.6%)	78 (2.2%)	127 (2.8%)	105 (2.9%)	21 (1.8%)
Dyslipidaemia	3428 (26.7%)	865 (24.5%)	1270 (27.8%)	1051 (29.4%)	242 (20.3%)
liver diseases	862 (6.7%)	224 (6.4%)	319 (7.0%)	260 (7.3%)	59 (4.9%)
Kidney diseases	1128 (8.8%)	238 (6.8%)	409 (8.9%)	371 (10.4%)	110 (9.2%)
Asthma	789 (6.1%)	116 (3.3%)	276 (6.0%)	302 (8.5%)	95 (8.0%)
Arthritis/rheumatism	4817 (37.5%)	1126 (31.9%)	1745 (38.2%)	1499 (42.0%)	447 (37.5%)
Digestive disease	3718 (28.9%)	1038 (29.4%)	1339 (29.3%)	1042 (29.2%)	299 (25.1%)

**Table 2.** Determinants of vaccination status among older adults.

Dep. Variable (n/mean)	Vaccination (at least one dose) vs. others		Completed primary series vs. others		Booster vs. others	
	n=12900	mean=.939	n=12900	mean=.913	n=12900	mean=.773

	OR (95% CI)	p value	OR (95% CI)	p value	OR (95% CI)	p value
Female	0.83 (0.70–0.98)	0.026	0.82 (0.71–0.94)	0.005	0.93 (0.86–1.01)	0.094
Married	1.52 (1.26–1.83)	0.000	1.40 (1.18–1.65)	0.000	1.19 (1.06–1.35)	0.004
Ethnic minorities	0.91 (0.64–1.29)	0.599	0.67 (0.52–0.88)	0.004	0.65 (0.54–0.78)	0.000
Urban Hukou	0.63 (0.51–0.77)	0.000	0.73 (0.61–0.88)	0.001	0.98 (0.86–1.11)	0.727
Age group (ref. 52–59)						
Age 60–69	1.02 (0.81–1.29)	0.879	0.90 (0.74–1.11)	0.332	0.86 (0.76–0.98)	0.023
Age 70–79	0.80 (0.63–1.03)	0.082	0.66 (0.53–0.81)	0.000	0.65 (0.56–0.75)	0.000
Age 80+	0.37 (0.28–0.49)	0.000	0.27 (0.21–0.35)	0.000	0.27 (0.23–0.32)	0.000
Education (ref. illiterate)						
Primary school (literate)	1.05 (0.86–1.28)	0.623	0.92 (0.77–1.08)	0.309	1.02 (0.91–1.14)	0.780
Middle and high school +	1.17 (0.91–1.49)	0.218	1.16 (0.94–1.43)	0.170	1.11 (0.97–1.27)	0.145
Region (ref. East)						
Central	1.07 (0.87–1.30)	0.531	1.27 (1.06–1.51)	0.008	1.14 (1.01–1.29)	0.040
West	1.50 (1.21–1.85)	0.000	1.37 (1.14–1.64)	0.001	1.27 (1.12–1.45)	0.000
Northeast	1.38 (0.99–1.93)	0.061	1.20 (0.90–1.60)	0.216	0.89 (0.73–1.09)	0.253
Functionally dependent	0.29 (0.24–0.35)	0.000	0.32 (0.27–0.37)	0.000	0.45 (0.40–0.51)	0.000
Chronic conditions						
Hypertension	0.84 (0.72–0.99)	0.037	0.81 (0.71–0.93)	0.002	0.86 (0.79–0.95)	0.002
Heart diseases	0.61 (0.51–0.74)	0.000	0.72 (0.61–0.84)	0.000	0.86 (0.77–0.96)	0.007
Stroke	0.75 (0.58–0.96)	0.023	0.78 (0.62–0.98)	0.033	0.75 (0.64–0.89)	0.001
Diabetes	0.79 (0.65–0.98)	0.029	0.76 (0.64–0.91)	0.003	0.86 (0.76–0.97)	0.014
Lung diseases	0.71 (0.57–0.88)	0.002	0.85 (0.70–1.02)	0.087	0.94 (0.82–1.08)	0.374
Cancer	0.25 (0.18–0.34)	0.000	0.28 (0.21–0.38)	0.000	0.40 (0.32–0.51)	0.000
Dyslipidaemia	1.25 (1.04–1.51)	0.020	1.21 (1.03–1.43)	0.023	1.13 (1.01–1.27)	0.028
liver diseases	1.02 (0.74–1.41)	0.898	0.93 (0.71–1.21)	0.578	1.01 (0.85–1.22)	0.880
Kidney diseases	0.81 (0.62–1.04)	0.101	0.86 (0.69–1.08)	0.209	1.07 (0.91–1.26)	0.412
Asthma	1.02 (0.76–1.38)	0.872	0.95 (0.74–1.23)	0.718	0.88 (0.73–1.06)	0.185
Arthritis/rheumatism	1.44 (1.21–1.72)	0.000	1.37 (1.18–1.59)	0.000	1.15 (1.04–1.27)	0.006
Digestive disease	1.16 (0.96–1.39)	0.117	1.22 (1.04–1.43)	0.013	1.20 (1.08–1.33)	0.000
Constant	22.71 (15.93–32.37)	0.000	17.84 (13.12–24.25)	0.000	4.67 (3.80–5.75)	0.000

Note: Multinomial logit regression. Standard errors clustered at the household level. RRR stands for relative risk ratio. Phase 1 is the early period when the vaccination had not been available for more than 6 months; Phase 2 is a later period between the summers of 2021 and 2022.

**Table 3.** Determinants of early, late and non-vaccination

	Phase 1: from Jan 2021 to summer 2021 vs. unvaccinated (base)		Phase 2: from summer 2021 to summer 2022 vs. unvaccinated (base)		Vaccination in Phase 2 vs. Vaccination in Phase 1 (base)	
	(1)		(2)		(3)	
Dep. Variable (3 choices)	n=9890		n=9890		n=9890	

	RRR (95% CI)	p value	RRR (95% CI)	p value	RRR (95% CI)	p value
Female	0.80 (0.67–0.95)	0.010	0.82 (0.68–0.99)	0.035	1.03 (0.93–1.13)	0.605
Married	1.65 (1.35–2.01)	0.000	1.38 (1.12–1.71)	0.003	0.84 (0.72–0.97)	0.020
Ethnic minorities	0.58 (0.40–0.84)	0.004	1.38 (0.97–1.96)	0.075	2.39 (1.92–2.97)	0.000
Urban Hukou	0.59 (0.47–0.73)	0.000	0.41 (0.32–0.52)	0.000	0.70 (0.59–0.84)	0.000
Age group (ref. 52–59)						
Age 60–69	0.93 (0.73–1.18)	0.530	2.03 (1.54–2.69)	0.000	2.20 (1.85–2.61)	0.000
Age 70–79	0.66 (0.51–0.85)	0.001	2.21 (1.66–2.95)	0.000	3.35 (2.78–4.03)	0.000
Age 80+	0.19 (0.14–0.26)	0.000	1.63 (1.18–2.25)	0.003	8.44 (6.65–10.72)	0.000
Education (ref. illiterate)						
Primary school (literate)	1.00 (0.82–1.23)	0.969	0.99 (0.80–1.22)	0.900	0.98 (0.86–1.12)	0.792
Middle and high school +	1.12 (0.87–1.44)	0.388	0.87 (0.66–1.14)	0.311	0.78 (0.66–0.92)	0.003
Region (ref. East)						
Central	0.88 (0.71–1.09)	0.231	0.97 (0.77–1.22)	0.802	1.11 (0.95–1.29)	0.207
West	1.38 (1.10–1.74)	0.006	1.59 (1.25–2.02)	0.000	1.15 (0.99–1.35)	0.070
Northeast	1.06 (0.75–1.49)	0.742	2.03 (1.41–2.92)	0.000	1.91 (1.51–2.43)	0.000
Functionally dependent	0.20 (0.17–0.24)	0.000	0.48 (0.40–0.59)	0.000	2.41 (2.08–2.79)	0.000
Chronic conditions						
Hypertension	0.78 (0.66–0.92)	0.004	1.01 (0.85–1.21)	0.874	1.30 (1.16–1.45)	0.000
Heart diseases	0.57 (0.47–0.70)	0.000	0.65 (0.53–0.80)	0.000	1.14 (0.99–1.31)	0.064
Stroke	0.59 (0.45–0.77)	0.000	0.91 (0.69–1.19)	0.475	1.53 (1.25–1.87)	0.000
Diabetes	0.74 (0.59–0.92)	0.008	0.90 (0.71–1.13)	0.372	1.21 (1.04–1.42)	0.014
Lung diseases	0.73 (0.58–0.92)	0.007	0.73 (0.58–0.93)	0.012	1.00 (0.85–1.18)	0.982
Cancer	0.20 (0.14–0.28)	0.000	0.44 (0.31–0.63)	0.000	2.20 (1.60–3.03)	0.000
Dyslipidaemia	1.25 (1.02–1.52)	0.029	1.12 (0.90–1.38)	0.316	0.89 (0.78–1.03)	0.111
liver diseases	0.91 (0.65–1.28)	0.585	0.93 (0.65–1.33)	0.681	1.02 (0.81–1.28)	0.867
Kidney diseases	0.72 (0.55–0.94)	0.018	0.71 (0.53–0.95)	0.021	0.99 (0.81–1.20)	0.910
Asthma	0.90 (0.65–1.24)	0.513	1.00 (0.72–1.39)	0.997	1.11 (0.88–1.42)	0.381
Arthritis/rheumatism	1.54 (1.29–1.86)	0.000	1.23 (1.01–1.49)	0.038	0.79 (0.71–0.89)	0.000
Digestive disease	1.22 (1.01–1.47)	0.042	1.05 (0.85–1.29)	0.651	0.86 (0.76–0.97)	0.018

**Table 4.** Textual analysis for reasons of non-vaccination among participants without receiving the COVID-19 vaccine.

Reasons for non-vaccination	Summer 2021 n=3436	Summer 2022 n=856
Having contraindications	30%	48%
Old, frail, or with specific health conditions	25%	21%
Access problems^*^	22%	18%
Concerns about the side effect or efficacy	6%	9%
Never heard of the COVID-19 vaccine	8%	6%
Low chance of being affected by COVID-19	3%	4%
Rejected by vaccinators (rather than the above reasons)	5%	2%
Unwilling to get vaccinated (rather than the above reasons)	4%	3%
Other^#^	6%	2%

* Access problems comprised the following reasons: vaccine sites were far from home or couldn’t be located; individual mobility; local communities did not arrange vaccinations; short supply; and vaccination was not free.

# Other comprised the following reasons: fear of injection, being occupied or absent from home, and vaccination had been scheduled at the interview time.

Note: Reason categories aggregated from 20 types (Appendix Table A4, pp 6). The sum of shares can be greater than 100% because the interviewees may answer more than one category of reasons.

## Data Availability

The fifth wave of CHARLS will be available within two years after fieldwork is finished.
