# Supplementary material for: COVID-19 non-vaccination among older adults in China: a nationwide survey based on the China Health and Retirement Longitudinal Study (CHARLS)
Source: Res Sq. 2022 Dec 19:rs.3.rs-2380496. Preprint. [Version 1] doi: 10.21203/rs.3.rs-2380496/v1 (PMC9810236; doi:10.21203/rs.3.rs-2380496/v1)
Supplement: Supplement 1 [file NIHPPRS2380496v1-supplement-1.pdf]

Supplementary Files

This is a list of supplementary files associated with this preprint. Click to download.

- 3Appendix1215.docx
- flatYZhaors.pdf
- flatYZhaoepc.pdf
